# Plasma Levels of IL-17, VEGF, and Adrenomedullin and S-Cone Dysfunction of the Retina in Children and Adolescents without Signs of Retinopathy and with Varied Duration of Diabetes

**DOI:** 10.1155/2013/274726

**Published:** 2013-11-18

**Authors:** Kornel Semeran, Przemysław Pawłowski, Łukasz Lisowski, Izabela Szczepaniak, Jerzy Wójtowicz, Sławomir Ławicki, Alina Bakunowicz-Łazarczyk, Artur Bossowski

**Affiliations:** ^1^Department of Pediatrics, Endocrinology, Diabetology with Cardiology Division, Medical University of Białystok, Ulica Waszyngtona 17, 15-274 Białystok, Poland; ^2^Department of Pediatrics Ophthalmology with Strabismus Treatment Unit, Medical University of Białystok, Ulica Waszyngtona 17, 15-274 Białystok, Poland; ^3^Scientific Circle at the Department of Pediatric Ophthalmology with Strabismus Treatment Unit, Medical University of Białystok, Ulica Waszyngtona 17, 15-274 Białystok, Poland; ^4^Department of Biochemical Diagnostics, Medical University of Białystok, Ulica Waszyngtona 15A, 15-269 Białystok, Poland

## Abstract

The study objective was to assess chosen biochemical parameters of blood and bioelectric function of the retina in patients with T1DM. The study group consisted of 41 patients with T1DM with no signs of diabetic retinopathy. The control group included 21 pediatric patients. We performed (1) S-cone ERG testing with retina response stimulation in both eyes at the luminance of 0.1, 0.2, and 0.5 (cd × s/m^2^) with the 440 nm blue flash and light application of the amber background (300 ph cd/m^2^, 495 nm wavelength), (2) anthropometric measurements, (3) biochemical investigations: IL-17, VEGF, and ADM by the ELISA method. A comparison of the ERG results with biochemical investigations indicates a likely correlation between the worsening of retinal bioelectric function and VEGF levels growing with diabetes duration. We showed a negative correlation between ADM and HbA1c and described possible causes of ADM reduction observed in subgroup I. We demonstrated the presence of bioelectric retinal dysfunction already before the diagnosis of diabetic retinopathy, which provides new possibilities in the diagnosis of preclinical chronic complications of diabetes. The changes observed in the levels of IL-17, ADM, and VEGF suggest their involvement in the diabetic pathogenesis of eye diseases.

## 1. Introduction

Due to the systemic character of diabetes, virtually any organ can become damaged. The vision system is one of the first body parts exhibiting microcomplications and dysfunction, which is an important issue in modern diabetology [1].

Over 90% of patients with type 1 diabetes (T1DM) of 15 years' and longer duration show features of retinopathy, whereas in type 2 diabetes (T2DM) approximately 5% are affected at the time of diagnosis [1, 2].

The scale of the problem is extremely large, as among the 2.5 million of the Polish diabetic population, the number of cases with diabetic retinopathy is estimated at 600,000 [3]. Thanks to the dynamic progress in the development of diagnostic methods and the use of modern appliances, diabetes is known to affect all the anatomical structures of the eyeball [4].

Apart from vascular changes, diabetes may also cause neurophysiological lesions [5, 6]. Functional changes of retinal neurons are detected by electro physiological tests, being a noninvasive and highly objectivediagnostic tool. Quick detection of retinopathy and immediate implementation of appropriate treatment reduce individual and social expenses and improve life quality [7–9].

While preparing the project, we were aware of the necessity to distinguish risk groups at an early stage undetectable by standard methods. Color vision disorders experienced by diabetic patients were already described in the 1980s [10] and as shown by the study [11], they have been found to affect 8.6% of diabetic patients [12].

Disorders have been noted in the formation and transmission of S-cone signals, which may be associated with their high sensitivity to hypoxia [12]. The dysfunction of the OPs on the ascending arm of the b-wave in full-field ERG responses has been emphasized. A relationship has been revealed between the reduction in the amplitude of the OPs and longlasting diabetic retinopathy, especially the proliferative one [13, 14]. However, very few studies have been concerned with functional alterations developing at an early stage, when no lesions can be seen in the fundus of the eye.

The study objective was to find out, using the S-cone ERG protocol, whether juvenile patients with T1DM, with no visible lesions in the fundus of the eye, and with normal optic disc morphology reveal retinal dysfunction. We also wanted to determine if the disease duration and metabolic control affect neurophysiological conduction in the outer layers of the retina.

In addition, we analyzed the concentration levels of ADM as a protective factor, VEGF as a known marker of hypoxia, and IL-17.

Reports in recent years have mainly concentrated on IL-17, emphasizing its proinflammatory nature and involvement of Th17 cells that produce it in the pathogenesis of diabetes and other endocrinopathies and allergic diseases. Apart from diabetes, elevated levels of IL-17 in the serum and affected tissues have been reported by patients with rheumatoid arthritis, psoriasis, multiple sclerosis, and systemic lupus erythematosus. Its proangiogenic activity is known from research on cancer immunology [15, 16].

## 2. Material and Methods

The study group consisted of 41 patients with T1DM. Two subgroups were distinguished as follows:

 subgroup I: 22 patients with diabetes of ≥ 5 ≤ 10 years' duration.
 subgroup II: 19 patients with diabetes lasting longer than 10 years.

The control group included 21 pediatric patients.  Table 1 presents the group profile.

The inclusion criteria for the diabetic group were age 8−18 years, normal routine eye test results (best corrected visual acuity, color vision, slit lamp examination, and fundus examination), and negative history of the present illness that might affect the function of the retina and optic nerve. The exclusion criteria included patients with chronic diseases, eye disorders that might affect the retinal function and optic nerve, and electrophysiological testing. The inclusion criteria for the control group were age 8–18 years, normal routine eye test result, and negative history of the present illness known to affect the function of the retina and optic nerve.

Thirty-two patients with T1DM (including 19 from subgroup I, 13 from subgroup II) and 8 from the control group underwent the following. (1) First is light adapted blue flash (440 nm wavelength) ERG with increasing flash intensity 0.1, 0.2, and 0.5 (cd × s/m^2^) on the constant amber background (300 ph cd/m^2^, 494 nm wavelength). The stimulus duration was 4 ms and 10 responses were averaged and repeated twice. The ERGs were recorded simultaneously from both eyes with the use of DTL electrodes placed in the lower fornix. The pupils were dilated and subjects were light-adapted for 10 min. All ERG tests were performed on Espion E^2^ system (Diagnosys, LLC, USA). They also underwent (2) biochemical investigations: HbA1c, lipid profile, IL-17, VEGF, and ADM, using the immunoenzymatic ELISA method (R&D and EiAAb kits). Laboratory tests were performed in the Department of Laboratory Diagnostics, Children's University Hospital in Białystok, during routine diagnostic procedures and in the laboratory at the Department of Pediatrics, Endocrinology, Diabetology with Cardiology Division, Children's University Hospital.

The study was approved by the Bioethics Committee, Medical University of Białystok. Parents and children were informed about the purpose and nature of the study. The parents gave a written consent, whereas the children expressed a spoken consent before examination.

## 3. Statistical Analysis

The statistical analysis was performed using the Statistica 10.0 software (Cracow, StatSoft). Deviation from normality was evaluated by Kolmogorov-Smirnov test. Data were expressed as the mean value  ±  SD. We used paired samples *t*-test for the continuous variables with normal distribution. The Wilcoxon test was employed for the continuous variables outside the normal distribution. Multiple linear regression and Spearman correlation analyses were used to verify the correlation. All probability values were two-tailed, and a value of *P* < 0.05 was considered statistically significant.

## 4. Results

### 4.1. Electrophysiological Tests

All patients included in the study had full far and near visual acuity and clear ocular media.

A comparison of the mean values in the study group (all diabetic patients) and control showed a statistically significant reduction in the amplitude of the S-cone ERG b-wave in the group of 64 eyes for the 0.2 cd × s/m^2^ (9.0 ± 6.0 *μ*V versus 13.0 ± 5.0 *μ*V; *P* = 0.01) stimulus and for the 0.5 cd × s/m^2^ (18.0 ± 8.0 *μ*V versus 24.0 ± 10.0 *μ*V; *P* = 0.0084) stimulus. No significant deviations were noted in the wave amplitude or latency prolongation for the S-cone ERG a-wave and b-wave.

In subgroup I (38 eyes), a statistically significant decrease was observed in the amplitude of the S-cone ERG b-wave for 0.2 cd × s/m^2^ (8.0 ± 6.0 *μ*V versus 13.0 ± 5.0 *μ*V; *P* = 0.0065) and 0.5 cd × s/m^2^ (17.0 ± 8.0 *μ*V versus 24.0 ± 10.0 *μ*V; *P* = 0.010).

I n subgroup II (26 eyes), a significant drop in the b-wave amplitude was found only for the 0.5 cd × s/m^2^ stimulus as compared to the control group (18.0 ± 8.0 *μ*V versus 24.0 ± 10.0 *μ*V; *P* = 0.048).

A comparison between subgroup I and subgroup II did not show any significant effect of diabetes duration on the amplitude and latency of the S-cone ERG a- and b-waves.  Table 2 presents statistically significant results.

### 4.2. Biochemical and Biometric Investigations

All diabetic patients had higher levels of TCh, LDL, TG, and IL-17 as compared to healthy subjects, that is (175.0 ± 37.0 mg/dL versus 158.0 ± 18.0 mg/dL; *P* = ns) (95.0 ± 29.0 mg/dL versus 84.0 ± 16.0 mg/dL; *P* = ns) (85.0 ± 51.0 versus 74.0 ± 35.0 mg/dL; *P* = ns) (17.0 ± 3.0 pg/mL versus 16.0 ± 2.0 pg/mL; *P* = ns), respectively. They also showed higher levels of SBP (114.0 ± 9.0 mmHg versus 110.0 ± 11.0 mmHg; *P* = ns) as compared to healthy children. The mean level of VEGF was significantly higher in diabetic patients, yet without statistical significance (362.0 ± 237.0 versus 273.0 ± 142.0 pg/mL; *P* = ns). A statistically significant finding was that ADM level in this group was lower than in the control (36.0 ± 27.0 versus 58.0 ± 32.0 pg/mL; *P* < 0.05).

A comparison between subgroup I and control revealed significantly lower ADM values (27.0 ± 22.0 versus 53.0 ± 29.0 pg/mL; *P* < 0.05). We observed higher levels of TCh, LDL, and TG (181.0 ± 47.0 versus 158.0 ± 18.0 mg/dL; *P* = ns) (97.0 ± 34.0 versus 84.0 ± 16.0 mg/dL; *P* = ns) (92.0  ±  69.0 versus 74.0 ± 35.0 mg/dL; *P* = ns), respectively. The values of SBP were increased as compared to healthy subjects (114.0 ± 10.0 versus 110.0 ± 11.0 mmHg; *P* = ns). VEGF was considerably higher in the subgroup patients, yet without statistical significance (379.0 ± 200.0 versus 273.0 ± 142.0 pg/mL; *P* = ns). The concentrations of IL-17 were similar (16.0 ± 2.0 versus 16.0 ± 2.0 pg/mL; *P* = ns).

A comparison between subgroup II and control showed significantly higher BMI values in diabetic patients (23.0 ± 4.0 versus 20.0 ± 3.0; *P* < 0.05). Previously, we noted unfavorable differences in lipid metabolism for TCh, LDL, and TG (169.0 ± 24.0 versus 158.0 ± 18.0 mg/dL; *P* = ns) (93.0 ± 24.0 versus 84.0 ± 16.0 mg/dL; *P* = ns) (78.0 ± 22.0 versus 74.0 ± 35.0 mg/dL; *P* = ns). Also in this subgroup, ADM appeared lower than in healthy subjects (39.0 ± 23.0 versus 58.0 ± 32.0 pg/mL; *P* = ns). A tendency of higher IL-17 and VEGF levels was confirmed in diabetic patients as compared to the control group (18.0 ± 4.0 versus 16.0 ± 2.0 pg/mL; *P* = ns) (342.0 ± 278.0 versus 273.0 ± 142.0 pg/mL; *P* = ns).

In multiple regression analysis, b-wave amplitude for the impulse 0.2 cd × s/m^2^ demonstrated a significant inverse relationship with VEGF (*r* ^2^ = 0.38, coefficient *β* = −0.25, *P* < 0.04) and LDL (*r* ^2^ = 0.38, coefficient *β* = −0.45, *P* < 0.00002).

In multiple regression analysis, b-wave amplitude for the impulse 0.5 cd × s/m^2^ yielded significant inverse findings with HbA1c (*r* ^2^ = 0.43, coefficient *β* = −0.42, *P* < 0.003) and LDL (*r* ^2^ = 0.43, coefficient *β* = −0.84, *P* < 0.00004). In multiple regression analysis, b-wave implicit time for 0.5 cd × s/m^2^ demonstrated significant inverse relationship with HDL (*r* ^2^ = 0.31, coefficient *β* = −0.25, *P* < 0.04) and positive relationship with IL-17 (*r* ^2^ = 0.31, coefficient *β* = 0.33, *P* < 0.008). Data are presented in Table 3.

## 5. Discussion

Numerous studies have confirmed the correlation between metabolic control and the incidence of diabetes-associated visual complications. Among them, Wisconsin Epidemiology Study, Berlin Retinopathy Study, the Early Treatment Diabetic Retinopathy Study, and the United Kingdom Prospective Diabetes Study have focused on the natural course of diabetic retinopathy [11, 17–19]. In 2003, Younis et al. investigated the annual and overall prevalence of any sign of diabetic retinopathy, maculopathy, and vision-threatening diabetic retinopathy in patients with T1DM and T2DM, who underwent screening tests [20]. There is evidence that vision-threatening diabetic retinopathy has a detectable occult stage or an early symptomatic phase.

The collected scientific data suggest that neurophysiological alterations in the retina are already present in the initial stages of diabetes [6, 21].

We confirmed the impact of the disease and poor metabolic control measured by the percentage of HbA1c on the responses obtained from the S-cone ERG prior to clinical symptoms of retinopathy. Diabetic children were found to show a significant reduction in the S-cone ERG b-wave amplitude with stimuli ≥ 0.2 cd × s/m², as compared to healthy subjects.

Previously, it was indicated that the alterations in the electric activity of the middle layers of the retina, that is, in bipolar cells, horizontal cells, and Muller's cells, were present in the course of diabetes [13, 21]. The S-cone ERG applied in our study shows the activity of the S-cones (blue-sensitive), which are characterized by the maximum sensitivity to the short-wavelength part of the light spectrum and account only for 10% of the whole population of cones and less than 1% of the entire population of all retinal receptors [13]. Data are available indicating selective damage to the transduction pathway that begins in the S-cone and leads to discrete disturbances in color recognition, called tritanopia, which has not been fully explained yet. It seems that the S-cones are particularly sensitive to hypoxia involved in the pathophysiology of diabetes-associated alternations. Apart from the impaired metabolism of retinal neurotransmitters, scientists also point at the role of enhanced premature apoptosis or defective tightness in the blood-retinal barrier. Plasma protein migration accompanying this disorder may have an impact on the degree of absorption and/or light dispersion, causing a reduction in light reception by cones. Moreover, some of these proteins, including the non-enzymatic glycosylated ones, absorb ultraviolet light [4, 6, 22–25].

The ERG findings are similar to those reported by McFarlane et al. [6] in the assessment of the b-wave amplitude. In contrast to the above, we found no a- wave or b-wave delay. However, even the reduction of photopic negative response of S-cone amplitude itself suggests functional alterations in the inner layers of the retina that attenuates the quality of the S-cone impulse.

The multivariate regression analysis shows a negative correlation between HbA1c and the height of b-wave amplitude, which is observed for the most intense light pulse (0.5 cd × s/m^2^). As indicated in Table 2, the b-wave amplitude values for the stimulus differ significantly in both groups of diabetic children in comparison with the control group. This suggests that the highest diagnostic value, in the search for early functional changes in the retina, pulses with intensity of at least 0.5 cd × s/m^2^.

Our results are consistent with the data reported by authors who have assessed bioelectric activities of the retina by means of other electrophysiological methods [26–31]. A direct comparison of the results is difficult due to the use of different equipment, protocols, and group sizes. However, the common conclusion that can be drawn from these studies is that the objective neurotransmission disorders can appear in diabetic patients who still have normal eye fundus.

A number of growth factors responsible for diabetic retinopathy have been described. They are known to be involved both in the initial and proliferative phases of the process. However, no major factor has been found responsible for the stimulation of neovascularization in diabetic eye disease. The current study indicates the key role of VEGF, whose increase is one of the exponents of endothelial dysfunction, leading to increased permeability of the blood- tissue barrier; hence, its former name is vascular permeability factor [32–34]. The regulation of VEGF gene expression is stimulated mainly by hypoxia [35, 36]. High level of VEGF has been noted in the vitreous humor in retinopathy patients and in many eye disorders presenting with local hypoxia and neovascularization [37–39]. We found a tendency to higher serum levels of VEGF in diabetic patients as compared to the control, irrespective of diabetes duration. It can be assumed that the increased level of VEGF reflects endothelial dysfunction which appears earlier than the structural changes observed by ophthalmoscopy within the vascular walls. Taking into consideration the data reported by Santilli et al., this hypothesis can be referred to as a broader aspect of diabetic complications [40]. This author performed a prospective observation, showing a correlation between serum VEGF and the risk of nephropathy in diabetic patients. He proved that the maintenance of the increased serum level of VEGF facilitates the identification of patients with normal arterial blood pressure and normoalbuminuria, who are predisposed to the development of permanent microalbuminuria later in life.

An interesting hypothesis can be derived from reports on the significant increase in VEGF in the immunocytochemical analysis of nonvascular eye cells in diabetic patients, even, like in our study, with no signs of retinopathy [41, 42]. Considering the bioelectric disorders in the S-cone ERG, this would suggest that retinopathy originates in retinal neurons and glial tissue and only later affects blood vessels.

Our observations in the context of the data already collected on VEGF allow a wider perspective as to the role of this protein in the pathogenesis of visual complications of diabetes. Further studies are necessary to precisely determine the mechanisms linking VEGF to retinopathy.

In the last years, ADM has been found to have a potentially beneficial effect on the hemodynamics and neurohormonal regulation of the circulatory system. This protein has been detected not only in the cells of the adrenal medulla, but also in other tissues including smooth muscle cells, vascular endothelium cells, and retinal pigment epithelial cells. The collected data indicate a link between ADM and pathophysiological processes in diabetes accompanied by elevated levels of ADM, especially in advanced complications [43–45]. On the other hand, increased ADM can be associated with reduced renal clearance of this protein in the development of nephropathy, even though it is metabolized in the pulmonary circulation [46, 47]. This peptide is thought to be engaged in antiregulatory mechanisms, by preventing vasoconstriction, increasing natriuresis, and inhibiting platelet aggregation. Hence, the hypothesis that the level of ADM reflects endothelial activation is additionally supported by a positive correlation observed between cAMP and ADM, a second line transmitter involved in the regulation of the circulatory system [48, 49].

In 1999, that is, 6 years after ADM identification, Taniguchi et al. [50] were the first to present the effect of ADM on the eye, especially ciliary body and cornea, and thus on the regulation of intraocular pressure. Pigment epithelial cells also produce and secrete ADM, which stimulates back the proliferation of these cells. Udono et al. [45] claimed that levels of ADM in the vitreous humor of patients with vitreoretinal proliferations were significantly higher in patients with proliferative diabetic retinopathy. A few years later, the same author proved in vitro that hypoxia increases ADM expression in the human pigment epithelial cell line and that the use of exogenous ADM increases survival of the cell line exposed to hypoxia [51], thus confirming a beneficial effect of this protein on retinal metabolism. The data suggest that ADM may be involved in the pathogenesis of diabetic retinopathy, especially that retinal arteries have been recognized as its uptake point [52]. Thus, our observation of decreased serum ADM in children with diabetes of less than 10 years' duration, especially in the context of a weaker response in the S-cone ERG and higher serum levels of VEGF, lipids, and higher blood pressure, seems surprising, even though a substantial increase in the level of ADM has been demonstrated only in patients burdened with overt retinopathy, microalbuminuria, or renal failure [48]. Our study, however, does not exclude a local increase in the level of ADM, for example, in eye tissues, before its rise in the serum; in patients with diabetes >10 years' duration, the level of ADM is already higher. Taking into account the negative correlation between ADM and HbA1c in the ≤10 years' subgroup, this is presumably caused by the protein consumption in unfavorable conditions of hyperglycemia. An alternative hypothesis suggests that increased ADM in diabetes is a late effect associated with progressing vascular endothelial dysfunction. It should be emphasized that our study group consisted of children, mainly adolescents, who were in the period of intensive hormonal changes, which undoubtedly affected the circulatory system.

The fact is that the level of ADM in diabetes undergoes changes even in the pediatric population and that their direction and significance require further investigations.

Still very few studies, mainly performed on the animal models of autoimmune diabetes, indicate a disorder in IL-17, which can have a significant impact on the course of the disease. Also, clinical studies based on human material indicate a serious disturbance in the cell population in T1DM. The few available studies concerning children with T1DM have shown an increased secretion of IL-17 [53, 54].

Therefore, in further elucidation of the importance of Th17 and IL-17 cells in T1DM, interesting may seem the studies that evaluate the role of IL-17 from an early clinical stage to a later period (after the end of remission) and thus assess the importance of the initiation and modulation of the inflammatory response and in consequence the effect on the disease and disclosure of complications [55]. It is believed that IL-17 may play a major role in the cytokine network regulating immune responses, inflammatory responses, or angiogenesis. It is possible that IL-17 is indirectly involved in these reactions through the effect on the expression of other more targeted cytokines. Research of Numasaki et al. [16] has shown that IL-17 is an angiogenesis mediator, acting directly on endothelial cells and through other lymphokines with angiogenic properties. This can be confirmed by the common direction of changes obtained in our study in the levels of IL-17 and VEGF.

Since IL-17 is involved in the pathogenesis of many diseases described above and their complications, its regulation provides new diagnostic, prognostic, and therapeutic potentials.

## 6. Conclusion

Since we found that the S-cone visual pathway is affected in adolescent patients with type 1 diabetes and without evidence of retinopathy, the S-cone ERG test may be a useful marker of early stage of inner retinal damage. Our results indicate that the short-wavelength abnormalities detected in T1DM before the onset of retinopathy originate in the retina.

The changes observed in the levels of ADM, IL-17, and VEGF support their possible involvement in the microvascular complications of diabetes. Different than expected results in ADM concentration in group I indicate that further in vivo studies are needed to clarify the role of ADM in this process.

We believe that our findings may help elucidate the mechanisms of retinopathy in order to protect the eyesight in young diabetic patients.

## Figures and Tables

**Table 1:** General profile of the study groups.

	Patients with diabetes > 10 years	Patients with diabetes ≤ 10 years	Whole study group	Control group
Number of patients	22	19	41	21
Age (years)	16.0 ± 1.0	13.0 ± 3.0**	14.0 ± 3.0	15.0 ± 2.0
BMI (kg/m²)	23.0 ± 4.0*	20.0 ± 5.0	21.0 ± 4.0	20.0 ± 3.0
Mean HbA1c (%)	9.4 ± 1.6*	9.3 ± 1.7^∗∗^	9.3 ± 1.7***	5.5 ± 0.3
Total cholesterol (mg/dL)	169.0 ± 24.0	181.0 ± 47.0	175.0 ± 37.0	158.0 ± 18.0
LDL-cholesterol (mg/dL)	93.0 ± 24.0	97.0 ± 34.0	95.0 ± 29.0	84.0 ± 16.0
HDL-cholesterol (mg/dL)	60.0 ± 14.0	65.0 ± 17.0	63.0 ± 16.0	59.0 ± 11.0
Triacylglycerol (mg/dL)	78.0 ± 22.0	92.0 ± 69.0	85.0 ± 51.0	74.0 ± 35.0
Systolic RR (mmHg)	113.0 ± 8.0	114.0 ± 10.0	114.0 ± 9.0	110.0 ± 11.0
Diastolic RR (mmHg)	69.0 ± 5.0	69.0 ± 6.0	69.0 ± 6.0	69.0 ± 11.0
IL-17 (pg/mL)	18.0 ± 4.0	16.0 ± 2.0	17.0 ± 3.0	16.0 ± 2.0
ADM (pg/mL)	39.0 ± 23.0	27.0 ± 22.0**	36.0 ± 27.0***	58.0 ± 32.0
VEGF (pg/mL)	342.0 ± 278.0	379.0 ± 200.0	362.0 ± 237.0	273.0 ± 142.0

Data are presented as mean ± SD.

*P < 0.05 as compared to control group (Student's *t*-test).

**P < 0.05 as compared to control group (Student's *t*-test).

***P < 0.05 as compared to control group (Student's *t*-test).

**Table 2:** Comparison of b-wave amplitudes in S-cone ERG of the eyes of patients in all study groups.

Patients with diabetes > 10 years	Patients with diabetes ≤ 10 years	Whole study group	Control group
b-wave amplitude (*μ*V) for the 0.2 cd × s/m^2^ stimulus			
10.0 ± 5.0	8.0 ± 6.0*	9.0 ± 6.0*	13.0 ± 5.0

b-wave amplitude (*μ*V) for the 0.5 cd × s/m^2^ stimulus			
18.0 ± 8.0*	17.0 ± 8.0*	18.0 ± 8.0*	24.0 ± 10.0

Data are presented as mean ± SD.

*P < 0.05 as compared to control group.

**Table 3:** Multiple linear regression analyses for b-wave amplitudes for 0.2 cd × s/m^2^, b-wave amplitudes for 0.5 cd × s/m^2^, and b-wave implicit time for 0.5 cd × s/m^2^ as dependent variables.

Dependent variable	*r* ^2^	Independent variable	Unstandardized coefficients		Standardized coefficients β	*t*	*P*
			*B*	Standard *B* error			
b-wave amplitude 0.2 cd × s/m^2^	0.38	Constant	14.69	7.24		2.03	0.0469
		VEGF	1.10	0,25	−0.25	−2.13	0.0376
		LDL- cholesterol	−0.13	0.03	−0.45	−4.03	0.0001
b-wave amplitude 0.5 cd × s/m^2^	0.43	Constant	−0.2	7.72		−0.03	0.9799
		HbA1c	−1.81	0.57	−0.42	**−**3.19	0.0022
		LDL- cholesterol	−0.35	0.08	−0.84	−4.53	0.0004
b-wave implicit time for 0.5 cd × s/m^2^	0.31	Constant	46.40	1.55		30.02	0.0000
		HDL- cholesterol	−0.01	0.01	−0.25	−2.21	0.0307
		IL-17	0.22	0.08	0.33	2.75	0.0079

## Abbreviations

ADM: Adrenomedullin
BMI: Body mass index
cAMP: Cyclic adenosine monophosphate
ERG: Electroretinogram
HDL: HDL-cholesterol
IL-17: Interleukin-17
LDL: LDL-cholesterol
OPs: Oscillatory potentials
SBP: Systolic blood pressure
S-cone: Short-wavelength cone
T1DM: Type 1 diabetes mellitus
T2DM: Type 2 diabetes mellitus
TCh: Total cholesterol
TG: Triacylglycerol
Th17: T-helper 17 cells
VEGF: Vascular endothelial growth factor.
